# Genetic parameters of color phenotypes of black tiger shrimp (*Penaeus monodon*)

**DOI:** 10.3389/fgene.2022.1002346

**Published:** 2022-10-03

**Authors:** Md. Mehedi Hasan, Herman W. Raadsma, Peter C. Thomson, Nicholas M. Wade, Dean R Jerry, Mehar S. Khatkar

**Affiliations:** ^1^ The University of Sydney, Faculty of Science, Sydney School of Veterinary Science, Camden, NSW, Australia; ^2^ ARC Research Hub for Advanced Prawn Breeding, Townsville, QLD, Australia; ^3^ CSIRO Agriculture and Food, Queensland Biosciences Precinct, St Lucia, QLD, Australia; ^4^ Centre for Sustainable Tropical Fisheries and Aquaculture, College of Science and Engineering, James Cook University, Townsville, QLD, Australia

**Keywords:** *Penaeus monodon*, cooked, uncooked, color, genetic parameters

## Abstract

Black tiger shrimp (*Penaeus monodon*) is the second most important aquaculture species of shrimp in the world. In addition to growth traits, uncooked and cooked body color of shrimp are traits of significance for profitability and consumer acceptance. This study investigated for the first time, the phenotypic and genetic variances and relationships for body weight and body color traits, obtained from image analyses of 838 shrimp, representing the progeny from 55 sires and 52 dams. The color of uncooked shrimp was subjectively scored on a scale from 1 to 4, with “1” being the lightest/pale color and “4” being the darkest color. For cooked shrimp color, shrimp were graded firstly by subjective scoring using a commercial grading score card, where the score ranged from 1 to 12 representing light to deep coloration which was subsequently found to not be sufficiently reliable with poor repeatability of measurement (*r* = 0.68–0.78) Therefore, all images of cooked color were regraded on a three-point scale from brightest and lightest colored cooked shrimp, to darkest and most color-intense, with a high repeatability (*r* = 0.80–0.92). Objective color of both cooked and uncooked color was obtained by measurement of RGB intensities (values range from 0 to 255) for each pixel from each shrimp. Using the “convertColor” function in “R”, the RGB values were converted to L*a*b* (CIE Lab) systems of color properties. This system of color space was established in 1976, by the International Commission of Illumination (CIE) where “L*” represents the measure of degree of lightness, values range from 0 to 100, where 0 = pure black and 100 = pure white. The value “a*” represents red to green coloration, where a positive value represents the color progression towards red and a negative value towards green. The value “b*” represents blue to yellow coloration, where a positive value refers to more yellowish and negative towards the blue coloration. In total, eight color-related traits were investigated. An ordinal mixed (threshold) model was adopted for manually (subjectively) scored color phenotypes, whereas all other traits were analyzed by linear mixed models using ASReml software to derive variance components and estimated breeding values (EBVs). Moderate to low heritability estimates (0.05–0.35) were obtained for body color traits. For subjectively scored cooked and uncooked color, EBV-based selection would result in substantial genetic improvement in these traits. The genetic correlations among cooked, uncooked and body weight traits were high and ranged from −0.88 to 0.81. These suggest for the first time that 1) cooked color can be improved indirectly by genetic selection based on color of uncooked/live shrimp, and 2) intensity of coloration is positively correlated with body weight traits and hence selection for body weight will also improve color traits in this population.

## Introduction

The black tiger shrimp (*Penaeus monodon*) is regarded as a luxury food commodity because of its elegant sensory properties and high nutritional value. For black tiger shrimp, henceforth “shrimp”, visual sensory properties of bright red color after cooking contribute to higher prices and greater consumer acceptance (Tume et al., 2009). These color properties are derived from carotenoids (e.g., astaxanthins). Carotenoids are primarily produced by the primary producers (e.g., photosynthetic plants and algal species) of the ecosystems, and like most other secondary consumers, shrimp obtain their carotenoid elements through their diet. The body color of shrimp is mainly regulated by astaxanthin, which mainly presents in the exoskeleton and in the surface of abdominal muscle beneath the exoskeleton (Menasveta et al., 1993; Boonyaratpalin et al., 2001; Tume et al., 2009). Astaxanthin remains in both free and esterified forms with fatty acids. Moreover, when carotenoid astaxanthin binds with protein, it can also be found as caroteno-proteins. In marine invertebrates such proteins cause a big change in the carotenoid light absorption spectrum to produce a range of bright coloration, e.g., purple, blue, red or green (Britton et al., 1982). This bright coloration becomes more apparent when the shrimp are cooked, since cooking causes the caroteno-protein complexes to dissociate, resulting in increased color brightness, e.g., the typical bright red coloration of cooked shrimp (Britton et al., 1982; Tume et al., 2009; Wade and Glencross, 2011).

Besides coloration, carotenoid astaxanthin has a role in various other important physiological functions in shrimp, including growth, reproductive competency, survival, disease, and stress resistance (Supamattaya et al., 2005; Paibulkichakul et al., 2008; Niu et al., 2014; Wade et al., 2017). A study by Niu et al. (2014) has shown that diet supplementation of astaxanthin significantly enhanced the growth and immunological competency in *P. monodon*. Similarly, shrimp fed with blue green algae (*Dunaliella* sp.) containing astaxanthin, resulted in higher weight gain, survival, resistance to white spot syndrome virus (WSSV) infection and greater tolerance to stress conditions (e.g., low dissolved oxygen) (Supamattaya et al., 2005). An increase in egg numbers and spermatozoa was associated with elevated levels of astaxanthin in *P. monodon* (Paibulkichakul et al., 2008). An extensive review of the role and function of carotenoids in crustacean aquaculture revealed that carotenoids are essential for overall growth, performance, and coloration in shrimp (Wade et al., 2017). Since the source of carotenoid astaxanthin for shrimp comes from dietary sources, e.g., natural algae or added carotenoid astaxanthin in the dietary pellets, the addition of pigmentation additives causes a substantial increase in production costs. Numerous studies have shown that there is both an environmental and a genetic component of pigmentation for shrimp and aquaculture species. Background color of the rearing tank/environment tends to cause significant variation in animal pigmentation, e.g., *P*. *monodon* reared in a black tank display darker coloration (Wade et al., 2012). The genetic basis for pigmentation has been confirmed for several aquaculture species (e.g., salmon, rainbow trout), where some individuals are genetically superior in absorbing, transporting and depositing carotenoid astaxanthin from feed. However, no such information is available for *P. monodon*.

*P. monodon* is currently the major aquaculture crustacean species farmed in Australia and economically, is the second most important species in the world with an economic value of USD 5.7 billion and a production base of 750,600 tons in 2018 (FAO, 2020). Studies have shown that its current economic value can be increased significantly by improving color phenotypes. For example, AU$2–4/kg can be added for dark red colored shrimp over pale colored ones (Tume et al., 2009). To study and implement body color traits in selective breeding programs, the phenotyping of the traits under selection must be straightforward, standardised, relatively low cost, and accurate. To date there is no established protocol for measuring body color phenotypes for *P. monodon*. Measuring body color phenotype is complex, with patterned banding and uneven distribution of pigmentation, when compared to other traits (e.g., body weight). Its measurement can be influenced by various external factors, e.g., frequent changing of lighting conditions can affect the visual assessment of the body color of the shrimp. Broadly, there are two means for color phenotyping, namely 1) chemical, and 2) physical measurement approaches. In the chemical measurement approach, the phenotyping is done indirectly by NIR (near-infrared reflectance) or directly through HPLC analysis by quantifying color-producing chemical components in the shrimp. However, chemical analysis is expensive and time consuming and requires destructive sampling of animals, although it provides an accurate measurement of color phenotypes. On the other hand, the physical approach involves either visual determination of color intensity using a standardized color card, or by using an instrumental measurement where a tristimulus colorimeter (chromometer) measures the reflectance of light from the subject (e.g., shrimp) compared to a background calibration plate. Color may then be expressed in the L*a*b* system, with L* measuring lightness, a* redness/greenness and b* yellowness/blueness (Robertson, 1977). Studies have shown that this instrumental measurement approach is potentially very useful in color phenotype studies in aquaculture species, as the value a* (intensity of redness) is linearly correlated with the carotenoid pigment content in salmon fish flesh (Christiansen et al., 1995).

Although the body color phenotype has direct economic benefit to the shrimp aquaculture industry, no study has been conducted so far for genetic improvement of this trait in this species. Establishing the genetic basis for variation in pigmentation in *P. monodon*, will help to identify brood stock with superior color phenotypes. This will reduce the need for adding dietary astaxanthin level and will ultimately increase the overall profitability in *P. monodon* aquaculture (De Carvalho and Caramujo, 2017). Moreover, it is unknown whether selection on body color traits would have any detrimental effect on other commercially important traits (e.g., body weight, body length). Furthermore, it is unknown whether uncooked shrimp color is genetically correlated to cooked color. This information is particularly important, i.e., if there is any positive association between these two traits, then there will be opportunity to select live/uncooked body color of shrimp to improve the cooked color phenotype. The aims of the study were therefore: 1) To evaluate the efficiency of subjective measurement of color phenotypes based on manual scoring and compare with instrumental color analysis in *P. monodon*, 2) to estimate the genetic basis of the pigmentation phenotypes, 3) assess genetic correlation among cooked, uncooked color and other economically-important morphological traits and finally, 4) compare genetic gains by direct and indirect selection in color phenotypes.

## Material and methods

### Origin of the study population

All progeny in this experiment were sampled from commercial cohorts of *P. monodon* raised by Seafarms Group Ltd., as described in Foote et al. (2019). Briefly, wild broodstock were sourced from the Northern Territory, Australia and transferred to a commercial hatchery at Flying Fish Point, Queensland, Australia. Broodstock maturation was conducted within indoor flow-through tank systems (density of 3 m^−2^ at 28°C ± 0.5°C) and broodstock were fed a commercial maturation diet. For each cohort, broodstock were allowed to mate naturally within the tanks, with any unmated females then artificially inseminated following industry practices. Since tracing of broodstock contribution could not be done on farm, all potential broodstock were genotyped and parentage analysis was utilised to determine the contributing parents retrospectively as detailed by Guppy et al. (2020). Females were spawned in communal spawning tanks and spawned eggs were transferred hatching tanks, and hatched nauplii were then transferred into 20,000 L larval rearing tanks (LRTs) at a density from 100 to 125 nauplii/liter, and reared on a commercial diet until 30 DOC. LRTs were then pooled and stocked into 4,000 m^2^ grow-out ponds and reared under commercial conditions at a density of 45 m^−1^ until harvest. Immediately pre-harvest ponds were sampled by random castnet. The current study population comprised of 55 sires and 52 dams. In total 67 full sib and half-sib families were produced across 838 progeny and stocked across seven ponds as shown in Supplementary Table S1. From post-larval stage to harvest, the growth periods ranged from 124 to 143 days across ponds. Throughout the grow-out period the key water quality parameters were recorded, including dissolved oxygen, temperature, pH and salinity (pond water quality parameter has been provided in Hasan (2022).

The genotyping method described by Noble et al. (2020) was employed to determine pedigree structure. A genotype-by-sequence (GBS) based single nucleotide polymorphism (SNP) genotyping method was used for the brood stocks (DArTSeq) (Sansaloni et al., 2011). This DArTSeq data set was used to derive a targeted 4 K DArTcap custom SNP panel (4,194 SNPs) for genotyping of the offspring (Guppy et al., 2020). CERVUS version 3.0.7 (Kalinowski et al., 2007) was used to perform family assignment, and Colony V2.0.6.4 (Jones and Wang, 2010) was employed to allocate offspring to the appropriate genetic group. When the parental information was missing, an arbitrary parental ID was given to each group.

### Phenotypic recording and characterization for genetic analysis

**FIGURE 1:**
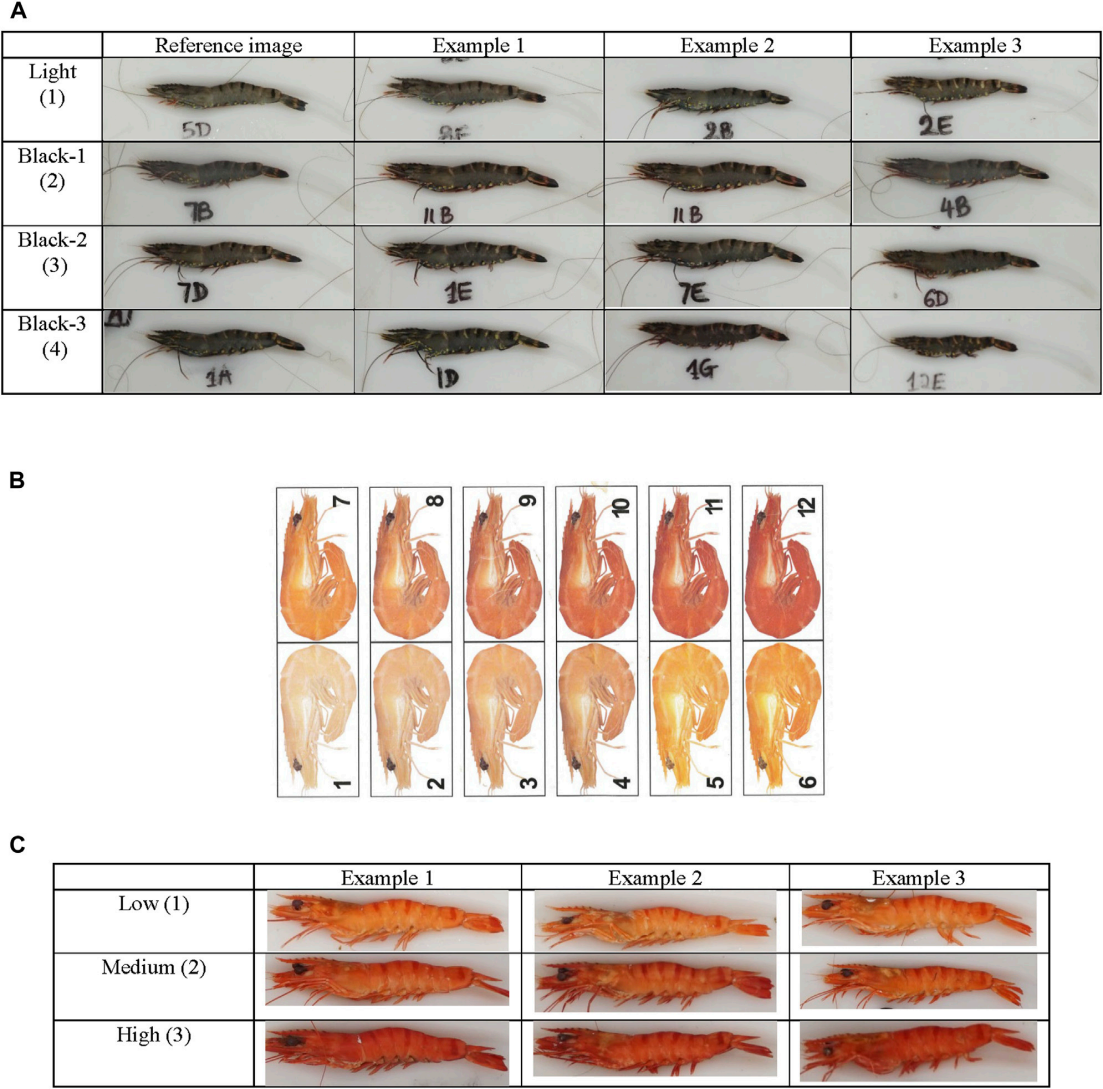
The reference image used for the scoring of uncooked and cooked shrimp. **(A)** Uncooked shrimp color chart, **(B)** commercial grade shrimp color score card (source: Aqua-Marine Marketing, Newport, QLD) and **(C)** cooked shrimp color chart.

The color of uncooked shrimp was subjectively scored by a single individual on a scale from 1 to 4, with “1” being the lightest/pale color and “4” being the darkest color (Figure 1A). For cooked color of the same shrimp, the shrimp were graded firstly by subjective scoring using a commercial grading score card, where the score ranged from 1 to 12 representing light to deep coloration (Aqua-marine Marketing Pty. Ltd., Kippa-Ring, Queensland, Australia) (Figure 1B). However, the repeatability (Pearson correlation coefficient) of color scoring was not considered sufficiently reliable using the commercial grading score card (*r* = 0.72) (Table 3). Therefore, by manual inspection of the shrimp images from brightest and lightest colored to the darkest and most color-intense cooked shrimp, three grades of colors (a scale of 1–3) were identified and selected as reference images for color grading of cooked shrimp for scoring (Figure 1C). The reliability of this new cooked color shrimp scoring was evaluated by examining confusion matrix tables and estimating repeatability of the manual scoring on a sample of 288 images (Table 3).

Objective color was measured by a standard digital photographs. The instrument returned RGB intensities (values range from 0 to 255) for each pixel from each shrimp sampled. Using the “convertColor” function in “R”, the RGB values were converted to L*a*b* (CIE Lab) systems of color properties. The CIE Lab color assessment system aligns more closely with human perception of color (Gómez-Polo et al., 2016; Ly et al., 2020). This system of color space was established in 1976, by the International Commission of Illumination (CIE). Here the “L*” represents the measure of degree of lightness, values range from 0 to 100, where 0 = pure black and 100 = pure white. The value “a*” represents red to green coloration, where a positive value represents the color progression towards red and a negative value towards green. The value “b*” represents blue to yellow coloration, where a positive value refers to more yellowish and negative towards the blue coloration.

### Statistical analysis

The manually scored color traits were considered as ordinal categorical variables and an ordinal logistic mixed model was employed to estimate variance components. This model considers three and four scores from each of cooked and uncooked shrimp, respectively. For each single observation in the data set, the model has the following form:Where *Y*_*ij*_ is the color score of the *i*th shrimp in the *j*th pond, and *k* is the (ordinal) score threshold, with *c* being the number of score points (*c* = 3 points for cooked color and *c* = 4 points for uncooked colors. θ_*k*_ is the intercept for each of the score points. Pond_*j*_ is a fixed effect and *a*_*i*_ is the polygenic random effect of the individual shrimp, linked to the pedigree, with $a=\left\{a_{i}\right\}$ and where $a∼N\left(0,\sigma_{A}^{2}A\right)$ where **A** is the numerator relationship matrix. This type of ordinal logistic regression is also known as the proportional odds model (Agresti, 2003). Heritability was calculated on a liability scale as follows:

$$h^{2}=\sigma_{A}^{2}/\left(\sigma_{A}^{2}+\pi^{2}/3\right)$$

where π^2^/3 is the liability residual variance and $\sigma_{A}^{2}$ is the variance estimate attributed to the additive genetic effects.

log_*e*_[P(*Y*_*ij*_ ≤ *k*)/P(*Y*_*ij*_ > *k*)] = θ_*k*_ + Pond_*j*_ + *a*_*i*_ , *k* = 1, … , *c*–1

The following linear mixed model was employed for estimating variance components for each of the growth traits and body color traits (i.e., L*_uncooked_, a*_uncooked_, b*_uncooked_, L*_cooked_, a*_cooked_ and b*_cooked_):

*y*_*ij*_ = *µ* + Pond_*j*_ + *a*_*i*_ + *ε*_*ij*_

where *y*_*ij*_ is the observation of individual *i* in pond *j*, µ is the mean and Pond_*j*_ is the fixed effect of the *jth* pond, *a*_*i*_ is the additive genetic effect, both terms as defined in the ordinal model, and ε_*ij*_ is the random error, assumed $N\left(0,\sigma_{e}^{2}\right)$ . Heritability was estimated as

$$h^{2}=\sigma_{A}^{2}/\left(\sigma_{A}^{2}+\sigma_{e}^{2}\right)$$

Genetic and phenotypic correlations among the traits studied were estimated using bivariate mixed models, of the form

$$\left(\begin{array}{l} y_{ij1}\\ y_{ij2} \end{array}\right)=\left(\begin{array}{l} \mu_{1}\\ \mu_{2} \end{array}\right)+\left(\begin{array}{l} {Pond}_{j1}\\ {Pond}_{j2} \end{array}\right)+\left(\begin{array}{l} a_{i1}\\ a_{i2} \end{array}\right)+\left(\begin{array}{l} \varepsilon_{ij1}\\ \varepsilon_{ij2} \end{array}\right)$$

with terms defined as in the above univariate model, and subscripts 1 and 2 indicating the pair of traits 1 and 2. As well as variance component estimates as outlined for the univariate models, covariances between trait pairs for additive genetic and residual effects were estimated. Phenotypic and genetic correlation estimates were obtained using these variance and covariance component estimates. As software is not available for bivariate ordinal-ordinal and linear-ordinal models, to estimate the genetic correlation among ordinal and numerical traits, Pearson correlations between the estimated breeding values (EBVs) were calculated (Calo et al., 1973).

Indirect genetic selection, i.e. correlated response in trait *y* with 1 standard deviation (SD) selection differential in trait *x*, was calculated from the following equation (Falconer and Mackay, 1996):

$$CR_{y}=r_{g}\times h_{x}\times h_{y}\times SD_{y}$$

where *SD*_y_ is the SD of trait “*y*”. The correlated response in trait “*y*” as a percentage of gain possible from direct selection for trait “*x*” is calculated as % of indirect selection (IS), the relative efficiency of correlated response (CR) in trait *y* when selection is applied on trait *x* as a percentage of gain possible from direct selection for trait *y*, i.e.,

$$\%IS=\frac{CR_{y}}{SD_{y}}\times 100=r_{g}\times h_{x}/h_{y}\times 100$$

Data analysis was performed in R v 4.1.0 (R Core Team, 2021), and the genetic analyses using the ordinal and linear mixed models (variance/covariance estimation, EBV calculation) were performed using ASReml-R 4.0 (VSNi) (Butler et al., 2017). Note that, the estimated breeding values (EBVs) are taken as the best linear unbiased predictions (BLUPs) of the *a*_*i*_ in both ordinal and linear models.

## Results

The mean, standard deviation, and coefficient of variation for body color, body weight and body length traits of shrimp at harvest are provided in Table 1. The average estimates of the traits studied are provided in Table 1. The description and distribution of the manually-scored color traits are provided in Table 2.

**TABLE 1:** Phenotypic means, standard deviations and co-efficient of variation of body weight, body length and objectively measured color related traits of shrimp before and after cooking.

Trait	*n*	Mean	SD	CV%	Min	Max
Body weight (g)	838	13.64	3.50	25.7	1.04	26.21
Body length (cm)	838	10.56	0.95	9.0	4.78	12.85
L*_uncooked_	838	23.84	3.82	16.0	14.96	37.71
a*_uncooked_	838	0.80	1.72	209	−3.95	6.64
b*_uncooked_	838	9.80	2.04	20.8	3.73	16.01
L*_cooked_	838	44.06	4.12	9.4	30.72	58.13
a*_cooked_	838	56.85	6.00	10.6	17.01	70.72
b*_cooked_	838	55.84	6.46	11.6	15.45	70.67

**TABLE 2:** Distribution of manual scores for cooked and uncooked shrimp from 838 animals.

Trait	Appearance scores			
	1	2	3	4
Uncooked	Light *n* = 29 (3.5%)	Black-1 *n* = 272 (32.5%)	Black-2 *n* = 424 (50.6%)	Black-3 *n* = 113 (13.5%)
Cooked	Light orange *n* = 92 (11.0%)	Medium orange *n* = 472 (56.3%)	Bright orange *n* = 274 (32.7%)	—

As shown in Table 3, the confusion matrix table revealed that the commercial image scale for grading shrimp body color is not suitable, as suggested by low repeatability (*r* = 0.72) of repeated scoring on a sample of 288 images. Instead, using images from current population-specific samples, provided more accurate estimates. This was supported by observing the repeatability analysis of color scoring. The repeatability analysis of scoring using the commercial shrimp cooked color chart and using the reference image developed in this study revealed that the scoring system developed in this study is more reliable (repeatability, *r* = 0.84) than the commercial image scale (repeatability, *r* = 0.72).

**TABLE 3:** Confusion matrix and repeatability of repeated measurement of cooked shrimp based on commercial color chart and the one derived for this study. The repeatability is indicated by the correlation (*r*) between the replicates (1 vs. 2, 1 vs. 3 and 2 vs. 3) for both color-scoring systems.

Commercial system scores					
Replicate 2					
		Score = 9	Score = 10	Score = 11	
Replicate 1	Score = 9	60	10	0	*r* = 0.78
	Score = 10	5	12	1	
	Score = 11	0	2	4	
Replicate 3					
		Score = 9	Score = 10	Score = 11	
Replicate 1	Score = 9	53	12	0	*r* = 0.71
	Score = 10	6	16	2	
	Score = 11	0	3	2	
Replicate 3					
		Score = 9	Score = 10	Score = 11	
Replicate 2	Score = 9	54	16	0	*r* = 0.68
	Score = 10	5	12	1	
	Score = 11	0	3	3	
cores derived in this study					
Replicate 2					
		Score = 1	Score = 2	Score = 3	
Replicate 1	Score = 1	18	6	0	*r* = 0.81
	Score = 2	7	82	1	
	Score = 3	0	18	56	
Replicate 3					
		Score = 1	Score = 2	Score = 3	
Replicate 1	Score = 1	20	5	0	*r* = 0.92
	Score = 2	3	101	2	
	Score = 3	0	2	55	
Replicate 3					
		Score = 1	Score = 2	Score = 3	
Replicate 2	Score = 1	18	6	0	*r* = 0.80
	Score = 2	5	83	2	
	Score = 3	0	19	55	

**FIGURE 2:**
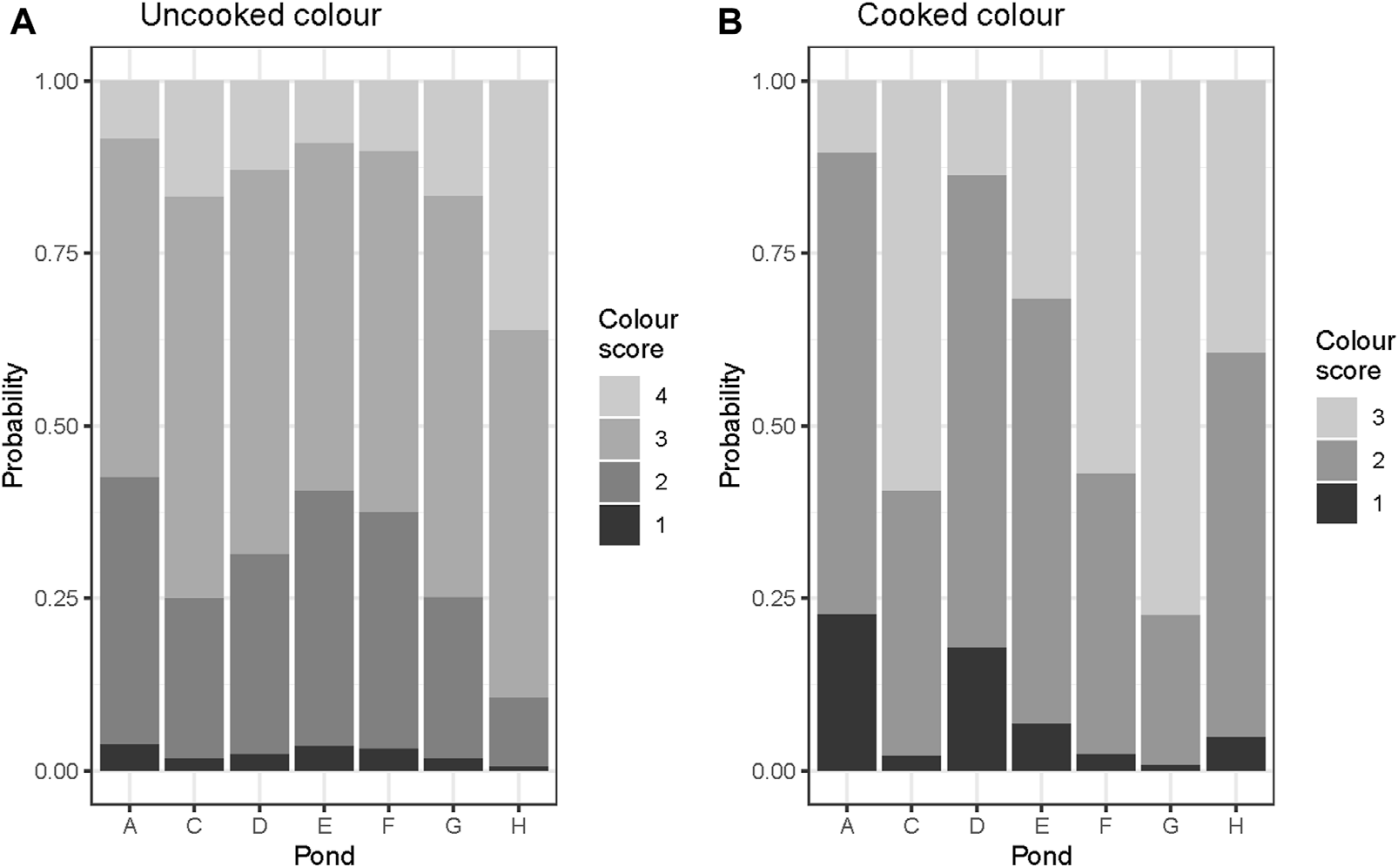
Probablility distribution of manual color scores by ponds **(A**–**G)**, **(A)** uncooked, and **(B)** cooked shrimp.

The observation of the probability distribution of color scores across ponds revealed that the bright cooked color shrimp were more common in ponds ‘C’, ‘F’ and ‘G’. In contrast, medium orange colored shrimp were abundant in ponds ‘A’, ‘D’ and ‘E’ (Figure 2B).

Heritability estimates for manually-scored uncooked and cooked body color traits were both 0.12 ± 0.04. Using instrumental measurement of body color phenotypes, heritability of the b* trait for uncooked color was highest at 0.35 ± 0.08, followed by a* trait for uncooked color at 0.29 ± 0.08, whereas they were generally low for cooked color indices. Details of these estimates are presented in Table 4.

**TABLE 4:** Genetic parameter estimates (±s.e.) for body color and body size traits.

Traits	*h* ^2^	σ^2^_*A*_	σ^2^_*e*_
Manual_uncooked_	0.12 ± 0.04	0.48 ± 0.19	π ^2^/3
Manual_cooked_	0.12 ± 0.04	0.46 ± 0.19	π ^2^/3
L*_uncooked_	0.14 ± 0.05	1.94 ± 0.83	11.44 ± 0.79
a*_uncooked_	0.29 ± 0.08	0.72 ± 0.22	1.71 ± 0.16
b*_uncooked_	0.35 ± 0.08	1.51 ± 0.44	2.75 ± 0.29
L*_cooked_	0.08 ± 0.04	1.06 ± 0.53	10.94 ± 0.66
a*_cooked_	0.05 ± 0.03	1.78 ± 1.17	30.92 ± 1.76
b*_cooked_	0.06 ± 0.03	2.03 ± 1.26	29.19 ± 1.72
Body weight	0.27 ± 0.07	3.95 ± 1.25	10.20 ± 0.91
Body length	0.32 ± 0.08	0.34 ± 0.10	0.69 ± 0.07

*h* ^2^ = heritability, σ^2^_*A*_ *=* additive genetic variance, σ^2^_*e*_ = environmental variance.

Strong genetic correlations were observed among several cooked and uncooked color traits (ranged from 0.82 to −0.88). Of these, strong positive genetic correlations were seen among L*_uncooked_ : L*_cooked_ (*r* = 0.82), similarly uncooked color score had a strong genetic correlation with cooked color score (*r* = 0.77). Of note is that L*_uncooked_ was also strongly correlated with both uncooked and cooked color (*r* = -0.88 and -0.81, respectively) suggesting this L*_uncooked_ could be a potential indirect selection marker for both cooked and uncooked color. Similarly, both L*_cooked_ and a*_cooked_ had strong genetic correlations with both cooked and uncooked color scores (Table 5), A medium strength genetic correlation was observed between body color and body weight traits (ranged, *r* = −0.39–0.43) as shown in Table 5. As expected, a correlation of near unity was observed for body weight and body length for both phenotypic (*r* = 0.93) and genetic (*r* = 0.96) correlations (Table 5).

**TABLE 5:** The genetic (lower diagonal) and phenotypic correlations (upper diagonal) among the studied traits.

	Manual (uncooked)	Manual (cooked)	L*_uncooked_	a*_uncooked_	b*_uncooked_	L*_cooked_	a*_cooked_	b*_cooked_	Body weight	Body length
Manual (uncooked)		0.44	−0.74	0.32	−0.28	−0.47	0.36	−0.04	−0.12	−0.12
Manual (cooked)	0.77		−0.57	0.28	−0.30	−0.62	0.59	−0.18	−0.03	−0.03
L*_uncooked_	−0.88	−0.81		−0.42	0.38	0.55	−0.53	0.00	0.05	0.06
a*_uncooked_	0.33	0.36	−0.25		0.09	−0.28	0.31	−0.04	−0.02	−0.04
b*_uncooked_	−0.41	−0.54	0.6	0.07		0.24	−0.20	0.25	0.11	0.11
L*_cooked_	−0.82	−0.84	0.82	−0.37	0.43		−0.51	0.33	0.15	0.16
a*_cooked_	0.74	0.81	−0.81	0.1	−0.53	−0.81		0.44	−0.04	−0.01
b*_cooked_	−0.35	−0.50	0.28	−0.63	0.30	0.48	−0.14		0.15	0.20
Body Weight	−0.28	−0.30	0.31	−0.21	0.15	0.43	−0.39	0.33		0.93
Body Length	−0.21	−0.2	0.24	−0.22	0.09	0.34	−0.27	0.32	0.96	

The computation of correlated response among the studied traits revealed that the selection on uncooked color with 1 SD selection intensity will lead to a correlated response of 77% on cooked color trait (Table 6). Similarly, selection in the uncooked color score will improve L*_cooked_ and a*_cooked_ values with an efficiency of 100% and 114% respectively. Likewise, selection on the L*_uncooked_ trait will lead to a response of 108.5% on the L*_cooked_ color trait and a 135% in the a*_cooked_ values (Table 6). Moderate indirect selection responses (range 13%–90%) were seen in all color traits, both uncooked and cooked, when selection for increased body weight is applied.

**TABLE 6:** Expected correlated response among color scores and color traits and body weight in both uncooked and cooked responses.

Trait 1	Trait 2	*h* ^*2*^ of trait 1	*h* ^*2*^ of trait 2	*r*_*g*_	Correlated response	IS %
Manual_uncooked_	Manual_cooked_	0.12	0.12	0.77	0.09	77.00
Manual_uncooked_	*a*_u_*_ncooked_	0.12	0.29	0.33	0.06	21.23
Manual_uncooked_	b*_uncooked_	0.12	0.35	−0.41	−0.08	−24.01
Manual_uncooked_	L*_cooked_	0.12	0.08	−0.82	−0.08	−100.43
Manual_uncooked_	a*_cooked_	0.12	0.05	0.74	0.06	114.64
Manual_uncooked_	b*_cooked_	0.12	0.06	−0.35	−0.03	−49.50
Manual_cooked_	L*_uncooked_	0.12	0.14	−0.81	−0.10	−74.99
Manual_cooked_	a*_uncooked_	0.12	0.29	0.36	0.07	23.16
Manual_cooked_	b*_uncooked_	0.12	0.35	−0.54	−0.11	−31.62
Manual_cooked_	L*_cooked_	0.12	0.08	−0.84	-0.08	−102.88
Manual_cooked_	a*_cooked_	0.12	0.05	0.81	0.06	125.48
Manual_cooked_	b*_cooked_	0.12	0.06	−0.50	−0.04	−70.71
L*_raw_	Manual_uncooked_	0.14	0.12	−0.88	−0.11	−95.05
L*_raw_	Manual_cooked_	0.14	0.12	−0.81	−0.10	−87.49
L*_raw_	L*_cooked_	0.14	0.08	0.82	0.09	108.48
L*_raw_	a*_cooked_	0.14	0.05	−0.81	−0.07	−135.54
L*_raw_	b*_cooked_	0.14	0.06	0.28	0.03	42.77
b*_raw_	Manual_uncooked_	0.35	0.12	−0.41	−0.08	−70.02
b*_raw_	Manual_cooked_	0.35	0.12	−0.54	−0.11	−92.22
b*_raw_	L*_cooked_	0.35	0.08	0.43	0.07	89.94
b*_raw_	a*_cooked_	0.35	0.05	−0.53	−0.07	−140.22
b*_raw_	b*_cooked_	0.35	0.06	0.30	0.04	72.46
a*_raw_	Manual_uncooked_	0.29	0.12	0.33	0.06	51.30
a*_raw_	Manual_cooked_	0.29	0.12	0.36	0.07	55.96
a*_raw_	L*_cooked_	0.29	0.08	−0.37	−0.06	−70.45
a*_raw_	a*_cooked_	0.29	0.05	0.10	0.01	24.08
a*_raw_	b*_cooked_	0.29	0.06	−0.63	−0.08	−138.50
Body weight	Manual_uncooked_	0.27	0.12	−0.28	−0.05	−42.00
Body weight	Manual_cooked_	0.27	0.12	−0.30	−0.05	−45.00
Body weight	L*_uncooked_	0.27	0.14	0.31	0.06	43.05
Body weight	a*_uncooked_	0.27	0.29	−0.21	−0.06	−20.26
Body weight	b*_uncooked_	0.27	0.35	0.15	0.05	13.17
Body weight	L*_cooked_	0.27	0.08	0.43	0.06	79.00
Body weight	a*_cooked_	0.27	0.05	−0.39	−0.05	−90.63
Body weight	b*_cooked_	0.27	0.06	0.33	0.04	70.00
Manual_uncooked_	Body weight	0.12	0.27	−0.28	−0.05	−18.67

*h* ^*2*^, heritabilty estimates; *r*_*g*_, genetic correlation; IS, indirect selection efficiency as % of direct selection response.

Figure 2 shows the distribution of the color scores obtained after fitting all other effects in the model. Distribution of uncooked color (Figure 2A) was more evenly distributed across ponds than cooked color (Figure 2B) suggesting a potential strong local environmental effect on cooked color.

## Discussion

Shrimp color is an economically important trait, associated with consumer acceptability, and must be accurately and inexpensively phenotyped for genetic evaluation (Blay et al., 2021). In most market scenarios, the price of shrimp is based on the color, both for cooked and uncooked states (Peshanoff and Jaensch, 2009; Wade et al., 2017). For cooked shrimp, bright colored individuals have the greatest market demand (Peshanoff and Jaensch, 2009). On the other hand, for uncooked shrimp, either pale or darker animals are preferred, depending on market demand based on socio-economic and cultural demographics (Mehar et al., 2020).

In our study we evaluated the cooked color phenotype of shrimp using both a commercially-used color chart and our own population-specific reference color chart. We found that the repeatability of color scoring is lower when using the commercial shrimp color chart. This may be due to the fact that the commonly available color scale may not match with our studied shrimp population. This suggests that the commercial shrimp color scoring chart may not be extensively applicable for color phenotyping of all the shrimp populations. For raw/uncooked shrimp color we found no standard reference color score chart for this species and like cooked color, we developed our own population-specific color chart for phenotyping. Overall, our findings suggest that, for subjective scoring of shrimp color phenotype, a population-specific reference color chart should be used for more accurate color scoring.

The genetic analysis of the body color traits revealed that there is sufficient genetic variation in these traits to include in a selective breeding program to improve these traits. There are several previous studies that have been conducted for determining heritability estimates of body color in shrimp species (Nguyen et al., 2014; Giang et al., 2019), however to the best of our knowledge, the present study is the first approach to estimate heritability of body color in *P. monodon*.

Heritability for manually scored body color traits were low (0.12 ± 0.04) for both uncooked and cooked color. These findings agreed well with studies with other aquaculture species for color traits (Rye and Gjerde, 1996; Kause et al., 2002). The low heritability estimates in our study for manually-scored color traits, may in part be attributed to the method of scoring. Subjective phenotyping scoring of color traits are subject to be influenced by the person who records it and measuring conditions (e.g., device used), and this can reduce the precision of the data recording. For example, in our current study repeatability of scoring (*r*) ranged from 0.82 to 0.92. Instead, there are numerous reports where increased heritability estimates could be achieved by objective phenotyping of color traits using colorimetric instruments. Although, heritability estimates of manually-scored color phenotypes were low, it should be noted that, this manual approach may be very useful when instrumental methods are not available (Gjedrem, 2010), in addition it is fast and efficient and could be done on a processing line without additional handling or preparation needed for colorimetric measurements.

The effect of rearing environment on shrimp body coloration is well established. For example, Tume et al. (2009) reported that rearing of shrimp (*P. monodon*) for just 28 days, either in black or white tanks, had significant effect on body coloration. The black tank reared shrimp were more bright orange in color than the white tank color reared one. Moreover, Alam et al. (2022) found that shrimp (*P. monodon*) reared in pond containing mangrove leaf litters had significant darker body coloration. Our study also revealed the variability of body coloration of *P. monodon* reared in different ponds, in particular cooked color, suggesting further study needs to be carried out to reveal the potential of genotype by environment (G×E) interaction for body color of this species.

From the six instrumental colorimetric measurements, heritability estimates ranged from 0.05 to 0.35. Similar to our findings, low to moderately high heritability value, ranging from 0.03 to 0.59, were also found in other shrimp species. Based on objective measurements of body color traits (e.g., lightness, yellowness and redness), Giang et al. (2019) reported heritability estimates ranging from 0.11–0.55, for body color traits of *Litopenaeus vannamei* reared in different environments. Nguyen et al. (2014) reported a heritability of 0.18 ± 0.05 and 0.08 ± 0.03 for uncooked and cooked color traits in banana shrimp (*Fenneropenaeus merguiensis*), respectively. In addition to shrimp, studies with other aquaculture species have shown that body color traits are heritable. Dufflocq et al. (2017), reported heritability estimates of 0.08 ± 0.02 and 0.04 ± 0.01 for flesh color traits, in two population of coho salmon (*Oncorhynchus kisutch*). For Atlantic salmon heritability estimates of 0.14 ± 0.03 (Tsai et al., 2015) and 0.07 ± 0.01 (Norris and Cunningham, 2004) were reported for color traits.

Of all the instrumental colorimetric measurements, heritability estimates were generally higher in the body color of the uncooked shrimp. For a*_uncooked_ and b*_uncooked_ values, the heritability estimates were 0.29 ± 0.08 and 0.35 ± 0.08, respectively, suggesting strong selection response will be obtained for these color traits. In particular, trait a*_uncooked_ can be a useful candidate trait for selection to improve redness color trait in shrimp, as previous studies have reported a linear relationship between carotenoid concentration and a* value in fish species, where carotenoid contents are the key contributor of bright coloration in crustaceans (Choubert, 1982; Skrede et al., 1990).

Surprisingly, heritability estimates were lower (ranged 0.05–0.08) for cooked color traits when body color phenotypes were measured instrumentally. This suggests that for genetic evaluation, instrumental measurements are more effective for uncooked body color traits than cooked traits. Given that both manually-scored and chronometrically-assessed body color traits identify significant additive genetic components, this suggests the higher potential of these traits for genetic improvement in *P. monodon*. Overall, our study confirms that the color phenotype has a substantial amount of genetic variation and is a good candidate trait for genetic improvement.

A key finding of our study was that the genetic correlation among the key body color traits were moderate to high, ranging from −0.88 to 0.81. Specifically, the genetic correlations between cooked and uncooked color traits were high (Table 3), suggesting that by selecting uncooked/live animals with desired color phenotypes, the cooked color of the animals can be improved genetically. This information is beneficial in shrimp breeding programs, as 1) it will eliminate the need for cooking the shrimp for phenotyping and thus will reduce overall associated costs in the breeding program; 2) will help breeders to preserve valuable genetic resource by not sacrificing them for cooked color phenotyping; and 3) measurements can be made at the same time as other important measurements are taken such as bodyweight and length due to moderately high genetic correlation between uncooked and cooked color of shrimp, it is predicted that selection for increased uncooked color (e.g., darker colored) will result in favorable change in cooked color (e.g., 77% at 1 SD selection intensity, Table 6) of shrimp.

Heritability of growth traits were moderately high in this study (e.g., 0.27 ± 0.07 for body weight and 0.32 ± 0.08 for body length traits), suggesting selection for these traits will lead to significant response in breeding programs. This finding corroborates with the previous findings, where heritability for growth traits were moderate to high, ranging from 0.23 to 0.69 in *P. monodon* (Hasan et al., 2020), indicating that these traits will be highly responsive during selection for genetic improvement. Of greater significance in this study is the impact of selection for growth on color traits. For the first time we show that color phenotypes were also moderate to highly correlated with growth traits in *P. monodon.* Similar finding was also reported by Giang et al. (2019) and Nguyen et al. (2014) for Pacific Whiteleg shrimp (*Litopenaeus vannamei*) and Banana shrimp (*Fenneropenaeus merguiensis*), respectively. Altogether these positive genetic correlations among color and growth traits suggests that a similar set of genes may be responsible for expression of these traits. Moreover, there might be physical linkage or pleiotropic effect and linkage disequilibrium among the underlying genetic mechanisms responsible for phenotypic expression of these traits (Falconer and Mackay, 1996). Similar findings of correlation with body color and growth traits have been reported in banana shrimp (Nguyen et al., 2014), salmonoids (Vieira et al., 2007) and in tilapia (Hamzah et al., 2016). The genetic correlations among body color and growth traits found from our study also indicate that there will be substantial correlated change when selection is applied on one trait or another. Selection for higher body weight will lead to a favorable increase of L*_cooked_ (108.48%) color of shrimp (Table 6). From a commercial perspective, this positive correlation between color and growth phenotypes suggests that color phenotype is highly suitable for shrimp aquaculture breeding program, since the growth combined with appealing coloration phenotype will increase overall profitability. Therefore, a selection index approach should be employed to simultaneously improve all the economically-important traits of the present population of shrimp, including growth, body shape and body color.

Studies with banana shrimp (Nguyen et al., 2014), salmon (Dufflocq et al., 2017) and tilapia (Hamzah et al., 2016), have also identified positive genetic correlations between body color and growth traits. This suggests that similar genetic and metabolic pathways may be involved in regulating the expression of color phenotypes in shrimp. However, gene(s) that control color traits in shrimp remain unknown. In general pigmentation in crustacean species is determined by carotenoid astaxanthin which is mainly ingested by food sources and then converted and stabilized as protein crustacyanin in the tissue. A number of studies have demonstrated that levels of carotenoids in shrimp are positively correlated with growth and survival, suggesting individual shrimp with superior color characteristics are capable of converting carotenoids from the feed more efficiently and they grow better. Our findings, coupled with other studies with shrimp species, clearly demonstrates that body color traits are sufficiently heritable in *P. monodon*. This suggests that this trait can be genetically improved, implying that some individuals of the population studied possess superior ability to convert the carotenoid astaxanthin and also have superior growth compared to others. Of significance is that parameters of uncooked color can improve cooked color based on either subjective scores or objective colorimetric measures (L*_uncooked_ ,a*_uncooked,_ b*_uncooked_). Finally, inclusion of cooked colour in the overall breeding objective for *P. monodon* will require clear economic benefits associated with the trait to be established through bio-economic modelling procedures as detailed by Marín-Riffo et al. (2021). Furthermore, all relevant genetic parameters and relative weights of all selection criteria, including uncooked colour and their relationship with the overall breeding objective will need to be established as detailed by Campos-Montes et al. (2017). However this is beyond the scope of the current study.

## Conclusion

Genetic parameter estimates for body color traits of *P. monodon* have been reported for the first time. The present study indicates that body color traits can respond effectively to selection. The high generic correlation between uncooked and cooked color scores indicates that, selection on dark colored (uncooked) shrimp will lead to enhanced intensity of cooked color. Moreover, positive genetic association among the growth and color traits indicates that, the selection for pigmentation and growth traits can be carried out simultaneously, without any unfavorable outcomes to these economically important traits. In summary, selective breeding can enhance growth and body color traits of shrimp simultaneously, thereby, helping to reduce the amount of food additives containing dietary astaxanthin. This will ultimately increase the overall product value and reduce feed costs.

## Data Availability

The original contributions presented in the study are included in the article/Supplementary Materials, further inquiries can be directed to the corresponding author.
