# Supplementary material for: Genetic parameters of color phenotypes of black tiger shrimp (Penaeus monodon)
Source: Front Genet. 2022 Oct 3;13:1002346. doi: 10.3389/fgene.2022.1002346 (PMC9573983; doi:10.3389/fgene.2022.1002346)
Supplement: Supplementary file 1 [file DataSheet1.PDF]

family	149	152	155	156	157	160	161	All_ponds
G1T1701_BR_002_G1T1701_BR_048	2		8					10
G1T1701_BR_002_G1T1701_BR_050			4	3				7
G1T1701_BR_003_G1T1701_BR_049	3	1	5	8				17
G1T1701_BR_005_G1T1701_BR_053	2	2	1	3				8
G1T1701_BR_006_G1T1701_BR_053	2	3	12	10				27
G1T1701_BR_007_G1T1701_BR_052	1							1
G1T1701_BR_008_G1T1701_BR_$4	4	30	5	5	36			80
G1T1701_BR_010_G1T1701_BR_$7					5	3	4	12
G1T1701_BR_012_G1T1701_BR_057			3	1				4
G1T1701_BR_014_G1T1701_BR_057			2					2
G1T1701_BR_015_G1T1701_BR_119	1			1				2
G1T1701_BR_017_G1T1701_BR_068			2	2				4
G1T1701_BR_018_G1T1701_BR_059		1						1
G1T1701_BR_023_G1T1701_BR_062	7	2	5	25	2	7	9	57
G1T1701_BR_024_G1T1701_BR_062	3		7	4				14
G1T1701_BR_030_G1T1701_BR_$1	2	5	1	2	9			19
G1T1701_BR_031_G1T1701_BR_064	1		1	1				3
G1T1701_BR_033_G1T1701_BR_064	2			3				5
G1T1701_BR_035_G1T1701_BR_065					1		5	6
G1T1701_BR_036_G1T1701_BR_065					3	1		4
G1T1701_BR_037_G1T1701_BR_067	4	1	3	14				22
G1T1701_BR_038_G1T1701_BR_067	3	2	2	8				15
G1T1701_BR_041_G1T1701_BR_170				1				1
G1T1701_BR_041_G1T1701_BR_461	1		1	3				5
G1T1701_BR_042_G1T1701_BR_139	7	4	4	16				31
G1T1701_BR_044_G1T1701_BR_198					2	3	6	11
G1T1701_BR_141_G1T1701_BR_192					1	7	7	15
G1T1701_BR_251_G1T1701_BR_123			2	1				3
G1T1701_BR_262_G1T1701_BR_113	3	1	5	5				14
G1T1701_BR_262_G1T1701_BR_131	7	22	16	42	13			100
G1T1701_BR_263_G1T1701_BR_166			10	16				26
G1T1701_BR_268_G1T1701_BR_403					1			1
G1T1701_BR_272_G1T1701_BR_$5	4		4	1				9
G1T1701_BR_283_G1T1701_BR_$3	1	1	1	1				4
G1T1701_BR_283_G1T1701_BR_095		1	2	1				4
G1T1701_BR_284_G1T1701_BR_362					5	7	13	25
G1T1701_BR_293_G1T1701_BR_401			4	1				5
G1T1701_BR_318_G1T1701_BR_360	6		7	12	2		3	30
G1T1701_BR_322_G1T1701_BR_060	1		3	1				5
G1T1701_BR_324_G1T1701_BR_119	4		4	3				11
G1T1701_BR_325_G1T1701_BR_102			1					1
G1T1701_BR_325_G1T1701_BR_402						15	17	32
G1T1701_BR_327_G1T1701_BR_$2				2				2
G1T1701_BR_359_G1T1701_BR_135					2	4	3	9
G1T1701_BR_363_G1T1701_BR_222		1			3			4
G1T1701_BR_367_G1T1701_BR_385					1			1
G1T1701_BR_428_G1T1701_BR_$6		3			3			6

G1T1701_BR_428_G1T1701_BR_211			2					2
G1T1701_BR_444_G1T1701_BR_055	1			2				3
G1T1701_BR_444_G1T1701_BR_134	3		6	4				13
G1T1701_BR_445_G1T1701_BR_$3			2	3				5
G1T1701_BR_445_G1T1701_BR_095	1	2	1	5				9
G1T1701_BR_450_G1T1701_BR_401	1		4	1				6
G1T1701_BR_|10_G1T1701_BR_211	2		9	4		1		16
G1T1701_BR_|11_G1T1701_BR_386	1		1	8				10
G1T1701_BR_|12_G1T1701_BR_187		1						1
G1T1701_BR_|13_G1T1701_BR_173		1			4			5
G1T1701_BR_|14_G1T1701_BR_391						3	2	5
G1T1701_BR_|1_G1T1701_BR_239	4	1	2					7
G1T1701_BR_|2_G1T1701_BR_131		1	3	5	2			11
G1T1701_BR_|3_G1T1701_BR_048	1		5	2				8
G1T1701_BR_|4_G1T1701_BR_049	3		1	1				5
G1T1701_BR_|5_G1T1701_BR_113	1			1				2
G1T1701_BR_|6_G1T1701_BR_231	2			1				3
G1T1701_BR_|7_G1T1701_BR_171			5	1				6
G1T1701_BR_|8_G1T1701_BR_360		2	14	4	6	12	5	43
G1T1701_BR_|9_G1T1701_BR_231				3				3
Total	91	88	180	241	101	63	74	838

838
